# Acquisition of Rater Agreement for the Stressful Life Events Schedule

**DOI:** 10.4172/2329-6488.1000124

**Published:** 2013-06-29

**Authors:** Lisa M James, Charles W Mathias, Bethany C Bray, Sharon E Cates, Sarah J Farris, Michael A Dawes, Nathalie Hill-Kapturczak, Donald M Dougherty

**Affiliations:** 1 Brain Sciences Center, Minneapolis Department of Veterans Affairs Medical Center, Minneapolis, MN, USA; 2 Division of Neurobehavioral Research, Department of Psychiatry, The University of Texas Health Science Center at San Antonio, San Antonio, TX, USA; 3 Methodology Center, Penn State University, State College, PA, USA

**Keywords:** Life stress, Addiction, Study methodology, Assessment, Consensus, Adolescents

## Abstract

Stress has been linked to a broad range of psychopathology including alcohol and drug dependence. Recent advances in our understanding of how stress interacts with biological systems involved in addiction has generated even greater interest in stress assessment among addiction researchers. The Stressful Life Events Schedule (SLES) capitalizes on the strengths and avoids the pitfalls of self-report checklist and interview-based stress assessments. Because the SLES depends on consensus ratings of a research team, this study examined rater agreement of stressful event ratings across the first year using the SLES. Individual ratings of stressful events were compared between two experienced and three new raters. Ratings were analyzed for life events generated from interviews of 70 adolescent psychiatric inpatients and 62 healthy adolescents. Inexperienced raters, with backgrounds in addiction research, reliably rated stressful events and rater agreement improved over a year’s time. Recommendations for successfully adopting the SLES for consensus rating are discussed.

## Introduction

Stress has been linked to a broad range of psychopathology including alcohol and drug dependence. Stress increases drug seeking behavior [1], risk for future alcohol and drug dependence [2], and vulnerability to relapses after drug treatment [3]. Recent advances in our understanding of how stress interacts with biological systems involved in addiction has generated even greater interest in stress assessment [4,5]. With this growing interest, members of the addiction research community are faced with adopting measures of stress to their assessment battery. The purpose of this manuscript is to describe the experience of 3 addiction researchers in learning to rate stress using an interview based procedure called the Stressful Life Events Schedule.

One of the major controversies in the stress assessment literature has been methodological debate on assessment procedure. The primary debate regarding stress measurement has centered on relative merits of self-report check lists versus interview-based measures. With self-report checklists, such as the Life Events Checklist [6], respondents note the occurrence or absence of specific stressful events that occurred within a designated time frame. Checklists are relatively inexpensive, quick to administer and score, and a number are designed specifically for certain developmental groups (e.g. adolescents; [7] for review). Depending on the testing situation and objectives of the study, the checklist method may be the most appropriate for assessing stress. However, checklists may not be optimal for studies requiring documentation of the timing/sequence of stressful events, the role of the individual in causing the event, or the contextual factors affecting the stressfulness of an event [7-10]. For example, in evaluating the stressfulness of an event with a checklist, each item is scored as having a particular stress magnitude regardless of the individual’s circumstances [8]. While this makes scoring simple, it can miscalculate how much stress the individual actually experienced. For example, endorsing the event “death of a friend” may reflect the death of one’s closest friend or someone the respondent has not seen in years. Presumably, these events would be associated with varying levels of stress, but a checklist cannot document these differences.

When more detailed information is needed about the context of a stressful event, interview-based methods may be a more appropriate measure. Perhaps the best recognized interview-based method is the Life Event and Difficulty Scale (LEDS; [11,12]; adapted for adolescents by [13,14]). The LEDS involves interviewing the individual about circumstances and timing of stressful events. Later, each event is rated by a team of experts as to (a) the stressfulness of events given the respondent’s history and current situation; (b), whether the event was caused by the individual; and (c) to whom the event occurred. While some consider interview-based methods the “gold-standard” because of the level of detail they measure [15] they are not above criticism: interviews are lengthy to administer and score. For example, the LEDS can require over 30 hours to complete the interview, write-up, and consensus rating [16]. Furthermore, the training required to learn how to use these instruments successfully is another significant time commitment. In standard epidemiological studies, researchers complete an initial eight days of training and periodic retraining to maintain consistency with ratings [16]. This time commitment can be cost- prohibitive for many addiction research testing situations.

Capitalizing on the strengths of both checklist- and interview-based methods, Williamson et al. developed the Stressful Life Events Schedule (SLES) for evaluating stressful events. The goal was to create an instrument that offers the efficiency of a checklist, but includes the level of detail of an interview [16]. Initial psychometric analyses indicate that the SLES appears to be a promising new tool for stress researchers. The SLES has demonstrated good concurrent validity with well-known checklist (e.g., Life Events Checklist) and interview-based methods (e.g., LEDS) and discriminant validity for both adolescents who are healthy or have a psychiatric disorder [16]. Furthermore, in contrast to typical interview-based stress assessments, SLES interviews and ratings can each be completed in less than 1 hour on average. While there is good evidence for both the validity and efficiency of the SLES, questions remain about how easily it may be adopted by new investigators.

While administration of the SLES is fairly simple for researchers familiar with semi-structured interviews, interpretations of the contextual factors involved in stressful events are inherently subjective. This raises the possibility of wide variance in stress rating, especially during initial experience with the rating system. Because the SLES depends on consensus ratings of stress event characteristics (like the LEDS and other interview-based measures), it is important to demonstrate the accuracy of rating for new raters. The purpose of this study was to examine the accuracy of SLES event ratings of addiction researchers across their first year of use of the SLES. It was expected that these raters initially would show low agreement with consensus ratings, but would improve over time. We were also interested in exploring how consensus reliability varied as a function of different types of stressful events.

## Method

### Measurement

The Stressful Life Events Schedule (SLES) [16] is composed of 77 items describing stressful events that may have occurred for the respondent or their significant others (e.g., parents, relatives, and friends). Events are grouped in nine primary categories: Crime, Deaths, Education, Health, Housing, Money, Romantic Relationships, Other Relationships, and Work. A tenth category (Additional Events) seeks to characterize other events that have affected the participant but are not included in one of the other event categories (e.g., the participant ran away from home).

The purpose of the SLES interview is to obtain information pertinent to three primary domains that are rated during the consensus conference: *Objective Threat, Behavior Dependence*, and *Focus* of event. *Objective Threat* refers to the amount of stress or unpleasantness that accompanies an event and is interpreted in the context of the individual’s developmental, cultural, and demographic characteristics. *Behavior Dependence* refers to the degree to which an event was directly caused or influenced by the individual’s behavior. Finally, *Focus* of the event refers to who or what is primarily involved in the event.

In the present study, information about stressful life events was obtained in a two-step process. First, participants completed a questionnaire version of the SLES where they identified which stressful events they had experienced, added events not included in the list, and rated the stressfulness of these events on a 4-point Likert-type scale where 1 = “Little or no effect” and 4 = “Great effect.” Second, these same participants were interviewed in a semi-structured format to gain more detail about the context of each event [16]. For example, if an participant indicated that her pet died, the interviewer would ask standard questions to ascertain more information (e.g., What happened? Was the death unexpected?) and the adolescent’s relationship to the pet (e.g., How long did you have the pet? How much time did you spend with your pet? How close were you to your pet?). These questions are aimed at eliciting information relevant to rating the three primary domains (*Objective Threat, Behavior Dependence*, and *Focus* of the event) during the consensus conference.

### Rating sample

SLES events were obtained through interviews of 132 adolescents: 70 psychiatric inpatients and 62 healthy controls. Adolescents where the focus of this study because this is the most common age range for onset of problem alcohol and/or drug use [17]. Both patients and controls were interviewed in this study in order to generate a sufficiently wide range of event types and scores. The mean age of the sample was 14.07 (*SD* = 1.5) years and 61% were girls. Most (68%) characterized their ethnicity as Hispanic and their race as predominantly White (43%) or More Than One Race (25%). There were no significant differences between the psychiatric inpatient and healthy adolescents in terms of age, gender, ethnicity, or race (*p*’s > .05). The most common diagnostic categories were Mood Disorders (*n*=49; 70%) and Disruptive Behavior Disorders (*n*=36; 51%) followed by Anxiety Disorders (n=18; 26%), Substance Use Disorders (*n*=9; 13%), and Psychotic Disorders (*n*=2; 3%); most met criteria for more than one diagnosis (median=2 diagnoses). Thirteen (18.6%) of the patients meet DSM-IV criteria for a substance use disorder (most frequently marijuana 14% and alcohol 4%). Control participants did not meet criteria for any psychiatric or substance use diagnosis. Written informed consent was obtained from all participants and all study procedures were reviewed and approved by the local Institutional Review Board.

### Raters

The new raters (Novice) included a senior-level behavioral psychologist (Experimental Psychology, Ph.D.), a mid-career addictions child and adolescent psychiatrist (M.D.), and a junior-level psychologist (Applied Biopsychology, Ph.D.). Two of the three Novice raters attended a 2-day SLES orientation session led by Dr. Williamson and the Expert raters, which included education on administration of the SLES interview and consensus rating procedures and practice. The experienced raters (Experts) were one bachelors-level and one masters-level research assistant who had conducted over 5,000 ratings under the supervision of Dr. Williamson for two years prior to this study.

### Consensus conference

In the consensus conference, an interviewer presents to the team a brief vignette, including a review of important relationships in the participant’s life. Each event description is then read aloud and rated by the consensus team in chronological order. For each event, the consensus team rated the events on the following three rating categories (Table 1).

Each event was rated independently of the previously rated events and, except for the interviewer, all members of the consensus team were blind to the participants’ subjective threat rating for the events. Once all members of the consensus team recorded their ratings, they stated their ratings for each of the three domains. When discrepancies occurred, the team discussed the ratings until a consensus was reached. To facilitate weighing the multiple factors that go into rating *Objective Threat*, a slide of each event was shown with examples of each possible score for that event; these examples were based on the Objective Threat Coding Logic created by Williamson et al. (2003) [16]. For the present study, rater agreement was examined across the first year of use of the SLES (corresponding to 1,425 rated events).

### Data analyses

**Figure 1:**
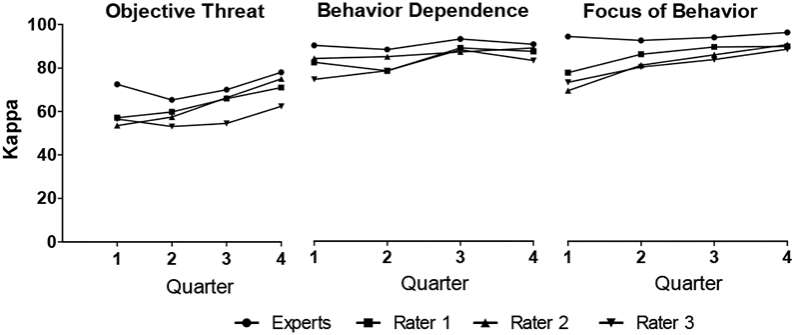
Rater agreement (kappa’s) between the final consensus rating and each individual Novice rater and the Experts for: *Objective Threat* (left), *Behavior Dependence* (middle), and *Focus* (right), shown in successive quarters of a year.

For all SLES events, each consensus team members’ ratings for *Objective Threat, Behavior Dependence*, and *Focus* of event were recorded, as was the final consensus team rating, and these values were analyzed using kappa to test rater agreement. Consistent with recommendations of [18] levels of agreement below 0.40 were considered poor agreement, values between 0.41 and 0.60 were considered moderate agreement, values between 0.61 and 0.80 were considered substantial agreement, and values greater than 0.81 were considered excellent agreement. To test the agreement as a function of time, kappa’s were analyzed in 3-month periods across the rating year (Figure 1). To test the agreement as a function of the different types of events, the kappa across all raters was calculated for all 10 SLES categories and the 10 most frequent event types reported by this sample (Table 2 and 3). The total number of events, the mean and standard deviation of Objective Threat, were reported for each event category. Repeated measures analyses of variance were used to test the change in kappa over each 3-month period for each domain. Univariate analyses of variance were used to compare kappa’s between the 10 different categories of events. These analyses were analyzed using PASW Statistics 19 (IBM Cooperation, Somers, NY).

## Results

### Rater agreement

Across the first year of ratings, there was moderate to excellent agreement between raters and the final consensus ratings of stressful life events. Table 2 shows the range of agreement (*k* = 0.56 – 1.00), which varied by both ratings domain but not event category. There was more agreement between raters and the consensus rating for *Behavior Dependence* and *Focus* domains than for *Objective Threat* (*F*_2,12_ = 28.3, *p*< .001).

When examining specific ratings within the 10 SLES categories of stressors (e.g. Crime, Education, etc), there were no significant differences in agreement between raters and the consensus rating for Objective Threat across the different Event Categories (*F*_9,40_ = 1.0, *p* = .432). The 10 most common events (in order of descending frequency) were analyzed for rater agreement on *Objective Threat*. For these most commonly endorsed events, the agreement ranged from moderate to substantial (0.53 - 0.76; Table 3).

### Rater agreement by quarter

General agreement improved across the first year of ratings (Figure 1). This was reflected in significant effect of rating period (i.e. Quarter) observed for *Objective Threat, Behavior Dependence*, and *Focus* (*F*_3,12_ = 7.3, 6.3, and 6.4, respectively). For *Objective Threat* there was there was significantly more agreement in the final (i.e., the fourth) quarter than in the third quarter (*p*<.001), which in turn had more agreement than the second quarter (*p*=.013). Note that the concordance at the first quarter was not significantly different than the concordance at any other quarter, likely due to a large standard error at the first quarter compared to the other quarters. Similarly for the *Focus* domain, there was there was significantly more agreement in the fourth quarter than at the third quarter (*p*=.029), which was had more agreement than the second quarter (*p*=.029). For *Behavioral Dependence*, agreement reached asymptote by the third quarter (3^rd^> 2^nd^ quarter *p*=.019, with no significant difference between 3^rd^ and 4^th^ quarters) (Figure 1).

## Discussion

This study examined rater agreement among addiction researchers across one year for events assessed using the Stressful Life Event Schedule (SLES). Novice raters rated stressful events with moderate to excellent accuracy compared to consensus ratings, and rater agreement improved over time. Some domains of stressful life events appeared easier to rate reliably than others. Rater agreement with the consensus ratings for *Behavior Dependence* and *Focus* were better than rater agreement for *Objective Threat*. However, the level of rater consensus agreement for *Objective Threat* was high. Importantly, it was similar to that in other reports which used more time-intensive objective threat rating procedures, characteristic of other stressful life event interviews [19,20].

*Objective Threat* may be expected to produce the least rater agreement, because raters mustconsider several factors to arrive at a rating. For example, raters may weigh how the participant’s age or developmental level at the time of the event might influence the level of threat. Also, if an event (e.g., crime or death) happened to someone other than the participant, raters must consider how close the participant was to that individual at the time of the event. The *Objective Threat Coding Logic* [16] that accompanies the SLES interview provides useful guidelines for raters and helps limit rater variability; however, in light of the unique factors that contribute to individual differences in stressful life events, the guidelines are not exhaustive and rater judgment is still required. Thus, discrepancies are expected to some extent and can be resolved during the consensus team meetings.

To facilitate the consensus process, our research group adopted procedures and rules to mitigate rater-drift and maintain efficiency. Other researchers interested in adopting the SLES may benefit from our experience and suggested guidelines for rating. Over the year, optimal accuracy and efficiency of the SLES rating process was best maintained by scheduling more frequent and shorter consensus meetings; weekly consensus meetings lasting about 1.5 hours were optimal.Longer meetings became less focused, slowed the speed of rating, and impeded the consensus resolution of ratings. If a substantial number of cases/events had accumulated from the previous week’s interviews, a second meeting was scheduled if needed. This approach was optimal for a five-person rating team; withmore or fewer raters, a different approach may be preferable. Generally speaking, the more raters, the longer it took to reach consensus.

The use of slideshows for reviewing coding logic facilitated the rating process. The *Objective Threat Coding Logic* [16] was included in the packet that two of the Novice raters received during their orientation to the SLES. However, repeated referencing within the packet was unwieldy. Thus, before the meeting, the interviewer created a slideshow with coding logic of just the specific events and sequence for the individual being rated. This was displayed during the reading of the vignette. This method provided visual cues for learning the coding logic and later helped prevent rater drift.

Several other procedures were established to facilitate the efficiency and harmony of the rating process. (1) The team refrained from asking questions until the interviewer had read the entire vignette. (2) When discrepancies arose during rating, they were debated in a fixed order: *Objective Threat*, then *Behavior Dependence*, and then the *Focus* of the event. This allowed raters to focus on the aspects of the event pertinent to the domain being rated and facilitated efficient consensus rating if discrepancies occurred across more than one of the three rated domains. (3) Each event for an individual case was rated independently of that person’s preceding events, which avoided conflating of threat ratings and allowed relatively “pure” ratings for each event. (4) Each rater was treated with equal authority in the consensus meeting, despite the diverse range of experience and workplace status within the team. While these procedures naturally emerged over time to resolve conflict and facilitate efficiency, we recommend new users of the SLES adopt these guidelines.

## Conclusion

Given the vast literature highlighting the association of stressors to addiction, there is a growing interest in identifying how particular stressful life events influence drug use and treatment those outcomes [21]. Researchers have sought to balance the level of detail necessary for accurately quantifying stress, while making the assessment process cost- and time-efficient. Depending on the testing situation, either checklist- or interview-based methods to quantify stress may be appropriate. However, the SLES provides a compromise; it is a fast and detailed method of stress assessment.

While the accuracy of the SLES depends on the rater agreement of stressful events, we found the rating processes were easily adopted by new raters with backgrounds in addiction research. The field of stress research has been criticized for “ongoing development of new tools without rigorous psychometrics” [7]. Prior analyses suggest that the SLES is a psychometrically sound tool that permits comprehensive assessment of stressful life events while minimizing time requirements [16]. The present study also provides evidence that the SLES is easily adopted by new investigators and requires minimal training. Furthermore, the present study extends findings from the original study of healthy controls and children/adolescents with mood and anxiety disorders [16] to provide evidence of good rater reliability for many events endorsed by adolescents with various forms of psychopathology. Recommendations for consensus ratings are described herein to facilitate adoption of the SLES by new researchers.

## Figures and Tables

**Table 1:** Stressful Life Event Schedule event categories and scales.

Rating Category	Scale
Objective Threat	
1 = Little or no effect	
2 = Some effect	
3 = Moderate effect	
4 = Great effect	
Behavior Dependence	
1 = Totally independent	
2 = Probably independent	
3 = Probably dependent	
4 = Totally dependent	
Focus	
1 = Self (event only involves participant)	
2 = Joint Focus (details of event involve participant and a friend/relative)	
3 = Other (event details involve somebody other than the participant)	
4 = Pet/Possession (details of the event involve a pet or possession)	

**Table 2:** Rater agreement for stressful event rating domains and categories.

Event Category	Total # events	Objective Threat Mean (SD)	Objective Threat K (SD)	Behavior Dependence K (SD)	Focus K (SD)
Education	245	1.58 (0.60)	0.64 (0.09)	0.75 (0.05)	0.52 (0.17)
Health	243	1.48 (0.57)	0.68 (0.03)	0.75 (0.04)	0.85 (0.05)
Non-Romantic Relationships	237	1.82 (0.68)	0.63 (0.09)	0.84 (0.04)	0.82 (0.13)
Death	168	1.76 (0.55)	0.60 (0.09)	0.67 (0.21)	0.90 (0.05)
Romantic Relationships	156	1.26 (0.46)	0.64 (0.11)	0.85 (0.08)	0.76 (0.12)
Housing	156	1.31 (0.45)	0.69 (0.05)	0.71 (0.11)	0.67 (0.22)
Work	98	1.29 (0.39)	0.64 (0.05)	0.83 (0.12)	0.80 (0.08)
Crime	55	1.75 (0.73)	0.74 (0.08)	0.96 (0.03)	0.86 (0.07)
Money/Possessions	38	1.34 (0.46)	0.67 (0.20)	1.00 (0.00)	0.52 (0.23)
Additional Events	29	1.49 (0.57)	0.56 (0.14)	0.73 (0.14)	0.60 (22)
Cumulative Events	1425	1.54 (0.60)	0.65 (0.09)	0.81 (0.08)	0.73 (0.13)

Note. K – average kappa agreement between raters and the final consensus

**Table 3:** Rater agreement for the ten most common stressful events.

Event	Total # events	Objective Threat K (SD)
I moved	95	.76 (.13)
My pet died or ran away	72	.65 (.04)
I had trouble with grades or school work	72	.64 (.13)
A close relative died	70	.56 (.16)
I changed schools	69	.64 (.17)
I started my menstrual cycle	61	.74 (.02)
I started dating someone or resumed a relationship	54	.58 (.13)
I was bullied at school or in my neighborhood	50	.53 (.11)
My close friend or family member was in the hospital or had an operation	50	.64 (.17)
I broke up with my boyfriend/girlfriend	50	.56 (.13)

Note. K – average kappa agreement between raters and the final consensus
